# Biochemical characterization of acyl-CoA:diacylglycerol acyltransferase2 from the diatom *Phaeodactylum tricornutum* and its potential effect on LC-PUFAs biosynthesis in planta

**DOI:** 10.1186/s12870-024-05014-7

**Published:** 2024-04-23

**Authors:** Sylwia Klińska-Bąchor, Kamil Demski, Yangmin Gong, Antoni Banaś

**Affiliations:** 1 Intercollegiate Faculty of Biotechnology, University of Gdańsk and Medical University of Gdańsk, Gdańsk, Poland; 2 Department of Plant Breeding, Swedish University of Agricultural Sciences, Alnarp, Sweden; 3 Oil Crops Research Institute of Chinese Academy of Agricultural Sciences, Wuhan, China

**Keywords:** DGAT, Omega-3 fatty acids, Eicosapentaenoic acid, *Nicotiana Benthamiana*, *Phaeodactylum tricornutum*, Plant biotechnology

## Abstract

**Background:**

Eicosapentaenoic acid (EPA) and docosahexaenoic acid (DHA), belonging to ω-3 long-chain polyunsaturated fatty acids (ω3-LC-PUFAs), are essential components of human diet. They are mainly supplemented by marine fish consumption, although their native producers are oleaginous microalgae. Currently, increasing demand for fish oils is insufficient to meet the entire global needs, which puts pressure on searching for the alternative solutions. One possibility may be metabolic engineering of plants with an introduced enzymatic pathway producing ω3-LC-PUFAs.

**Result:**

In this study we focused on the acyl-CoA:diacylglycerol acyltransferase2b (*Pt*DGAT2b) from the diatom *Phaeodactylum tricornutum*, an enzyme responsible for triacylglycerol (TAG) biosynthesis via acyl-CoA-dependent pathway. Gene encoding *Pt*DGAT2b, incorporated into TAG-deficient yeast strain H1246, was used to confirm its activity and conduct biochemical characterization. *Pt*DGAT2b exhibited a broad acyl-CoA preference with both di-16:0-DAG and di-18:1-DAG, whereas di-18:1-DAG was favored. The highest preference for acyl donors was observed for 16:1-, 10:0- and 12:0-CoA. *Pt*DGAT2b also very efficiently utilized CoA-conjugated ω-3 LC-PUFAs (stearidonic acid, eicosatetraenoic acid and EPA). Additionally, verification of the potential role of *Pt*DGAT2b *in planta*, through its transient expression in tobacco leaves, indicated increased TAG production with its relative amount increasing to 8%. Its co-expression with the gene combinations aimed at EPA biosynthesis led to, beside elevated TAG accumulation, efficient accumulation of EPA which constituted even 25.1% of synthesized non-native fatty acids (9.2% of all fatty acids in TAG pool).

**Conclusions:**

This set of experiments provides a comprehensive biochemical characterization of DGAT enzyme from marine microalgae. Additionally, this study elucidates that *Pt*DGAT2b can be used successfully in metabolic engineering of plants designed to obtain a boosted TAG level, enriched not only in ω-3 LC-PUFAs but also in medium-chain and ω-7 fatty acids.

**Supplementary Information:**

The online version contains supplementary material available at 10.1186/s12870-024-05014-7.

## Introduction

For many years, attention of scientists has been focused on engineering oilseed plants being able to produce and accumulate long chain polyunsaturated fatty acids (LC-PUFAs). Among them, especially important are representatives of ω-3 fatty acids – eicosapentaenoic acid (EPA; 20:5^Δ5,8,11,14,17^) and docosahexaenoic acid (DHA; 22:6^Δ4,7,10,13,16,19^). These fatty acids are an essential dietary supplement for animals and humans, as inter alia they maintain the proper functioning of the brain, nervous system and maintain cognitive functions [1]. Unfortunately, mammals are not able to produce them at a sufficient level covering their dietary requirements, therefore they must be supplemented exogenously. Currently, the main source of ω-3 fatty acid consumption is marine fish, although their primary producers are marine microalgae. Therefore, they are a potential source of enzymes that may allow for successful production and accumulation of ω-3 fatty acids *in planta*. The most important enzymes are native desaturases and elongases, because their sequential action leads to production of LC-PUFAs. Till now, quite many of them (from various marine organisms) have been identified, characterized and their potential role in engineering of plants to produce ω-3 fatty acids has been confirmed [2–7]. Nevertheless, other promising enzymes are still being sought after, especially those supporting their accumulation in the triacylglycerol (TAG) pool, since this lipid class dominates in plant oilseeds.

Photosynthetic microalgae synthesize significant amounts of LC-PUFAs, which are mainly accumulated in galactolipids. They constitute only a minor pool of the triacylglycerol fraction. However, under stress conditions such as nitrogen- or phosphorus-depletion, they can turn their metabolism toward TAG production [8, 9]. Biosynthesis of TAG can be performed via multiple pathways. The first one is known as Kennedy pathway and is acyl-CoA-dependent [10]. Membrane-bounded enzymes catalyze sequential conjugation of an acyl group to the glycerol backbone. Firstly, glycerol-3-phosphate acyltransferase (GPAT) catalyzes the transfer of an acyl group from acyl-CoA to glycerol-3-phospahte (G3P) and forms lysophosphatidic acid (LPA), which is further converted into phosphatidic acid (PA) via acyl-CoA:lysophosphatidic acid acyltransferase action (LPAAT). Next step is dephosphorylation of PA leading to diacylglycerol (DAG) production. Final step of TAG production via Kennedy pathway is action of acyl-CoA: diacylglycerol acyltransferase (DGAT) responsible for transfer of an acyl group from acyl-CoA pool into the *sn*-3 position of glycerol backbone of DAG. In parallel, acyl-CoA-independent reactions mediating TAG biosynthesis can occur. One of them is transfer of an acyl group from the *sn*-2 position of phosphatidylcholine to the *sn*-3 position of diacylglycerol, governed by phospholipid:diacylglycerol acytransferase (PDAT); [11]. The second one is mediated via DAG:DAG acyltransferase (DGTA) which utilized two DAG molecules - acyl donors and acceptors to form TAG [12]. In eukaryotes, the activity of all three described mechanisms of final TAG production has been confirmed, however, only genes encoding DGAT and PDAT enzymes have been identified.

Knowledge about microalgae PDAT enzyme and its participation in ω-3 and ω-6 fatty acids accumulation in TAG species remains deficient. This enzyme was identified and characterized in microalgal species such as *Chlamydomonas reinhardtii* and *Myrmecia incise* [13, 14]. The DGAT enzymes – DGAT1s, DAGAT2s and DGAT3s have been characterized already inter alia for *C. reinhardtii, Ostreococcus tauri, Phaeodactylum tricornutum* or *Nanochloropsis* [15–20]. All three identified DGATs have similar function but little sequence homology and exhibit different substrate specificity.

Among mentioned organisms, *P. tricornutum* represents the best studied diatom, which plays important role in suppling marine primary productivity. Moreover, due to the availability of its completely sequenced genome [21], well developed methods of genome editing, fast growth rate and ease of cultivation, *P. tricornutum* has become a model diatom. This organism is also able to produce abundant intracellular TAGs under certain conditions and to produce EPA reaching 30% of the total fatty acids, parallel with production of other LC-PUFAs fatty acids intermediates in trace level [8, 9, 22]. All of these make *P. tricornutum* a great model organism for studies on LC-PUFAs biosynthesis and accumulation. All three *Pt*DGATs (DGAT1, DGAT2 and DGAT3) residing in the chloroplast endoplasmic reticulum and can utilize both ER- and chloroplast-derived diacylglycerol [23]. *Pt*DGAT2 seems to be the most promising in further research on the *in planta* incorporation of the LC-PUFAs biosynthetic pathway. Overexpression of different isoforms of *Pt*DGAT2 in *P*. *tricornutum* led to production of elevated overall lipid content and increased the levels of LC-PUFAs, especially EPA or DHA [24–26].

It is postulated that, the efficient metabolic engineering of ω-3 LC-PUFAs in oilseeds, may depend on DGAT enzymes from microalgae, which may mediate the flux of non-native fatty acids to TAG and thus solve the problem of the often-encountered bottleneck – the efficient production and accumulation of ω-3 fatty acids *in planta* [7]. Therefore, we decided to verify whether co-expression of DGAT enzyme from *P. tricornutum* with gene combinations aimed at the production of EPA will result in increased accumulation of this fatty acid in the TAG pool. To answer this question, we used *Pt*DGAT2b, which effectiveness in elevated TAG production was confirmed in yeast and *P. tricornutum* [19, 26]. The experiments were conducted via transient expression in *Nicotiana benthamiana* leaves to point out further hypothetical potential in transgenic oilseed plant production. Additionally, we conducted a broad radiolabeled in vitro biochemical characterization of *Pt*DGAT2b, including optimization and determination of substrate specificity toward different diacylglycerols and various acyl-CoAs (ranging from medium chain fatty acids to LC-PUFAs) of *Pt*DGAT2b.

## Results

### Complementation of TAG synthesis in yeast and optimization of assays implemented in *Pt*DGAT2b study

The coding sequence of *Pt*DGAT2b was expressed in the TAG-deficient quadruple mutant strain of *Saccharomyces cerevisiae* H1246. This yeast strain possess deletion in four genes engaged in storage lipid synthesis: ARE1, ARE2, DGA1 and LRO1 [27]. Consistent with previous study done by Gong et al. [19], a spot corresponding to TAG was observed (Figure S1). Additionally, preliminary in vitro experiments with [^14^C]-labeled acyl-CoA and unlabeled diacylglycerol, confirmed *Pt*DGAT2b’s ability for TAG biosynthesis (Figure S2).

**Fig. 1:**
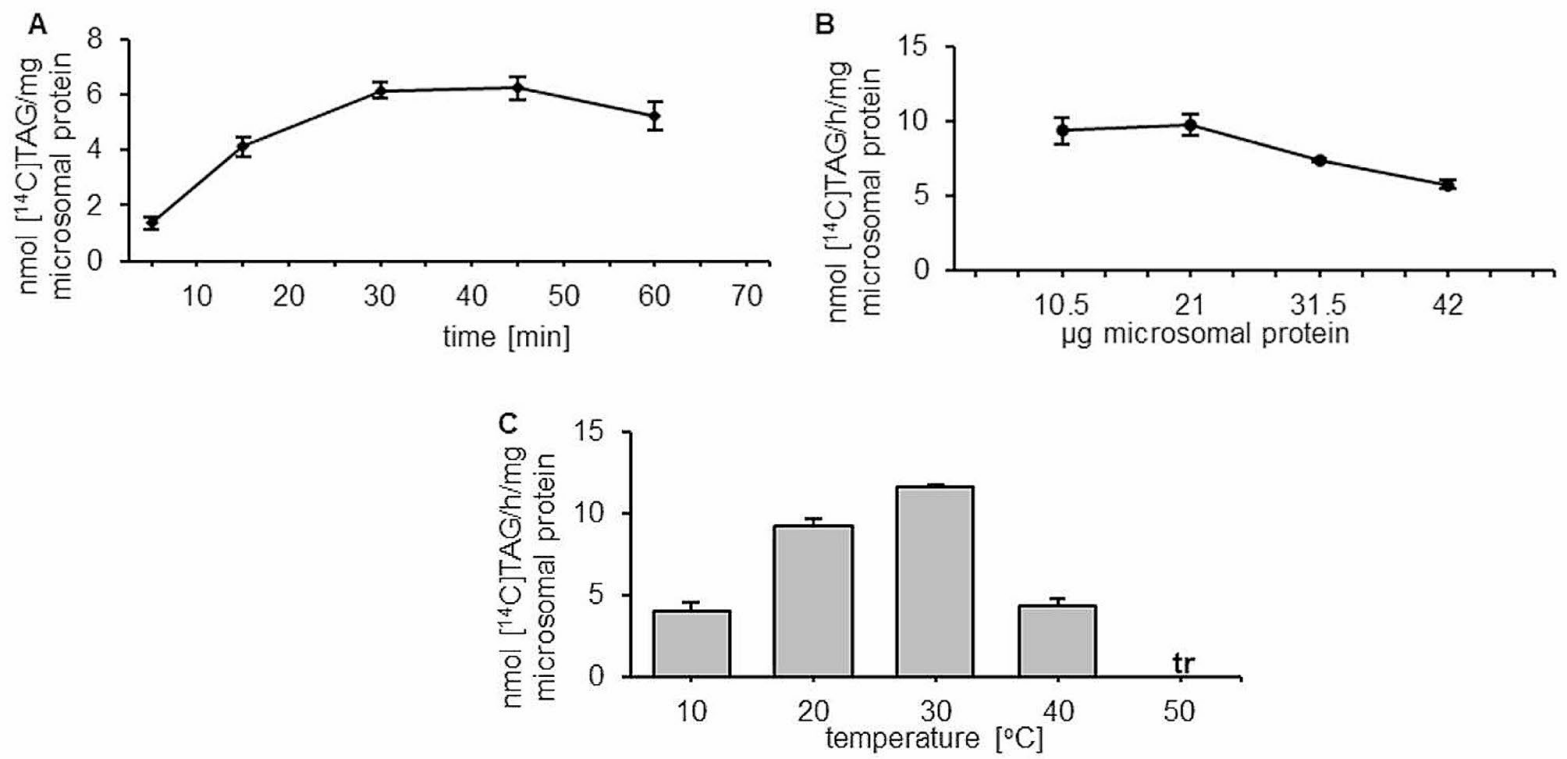
Optimization of the activity of acyl-CoA: diacylgycerol acyltransferases – DGAT2b from *Phaeodactylum tricornutum.* Effects of various factor on enzyme activity: (**A**) – reaction time; (**B**) – amount of microsomal protein; (**C**) – temperature. Error bars present standard deviations between independent biological replicates

In the present study we conducted in vitro experiments determining the biochemical properties of this enzyme. Firstly, the optimization assays were conducted and the effects of three different parameters affecting *Pt*DGAT2b in vitro activity were tested: reaction time, the amount of microsomal protein and reaction temperature. The results showed that the rate of the reaction catalyzed by *Pt*DGAT2b was linear almost up to 30 min of incubation, longer reaction time resulted in a decline in activity (Fig. 1A). Aliquotes of microsomal fraction ranging from 10.5 µg to 21 µg of microsomal protein were optimal for the assays, nevertheless for better visualization of synthesized TAG spots the higher microsomal protein content was used in subsequent reactions. Further increase of added microsomal fraction led to reduced enzyme activity (Fig. 1B). The optimal reaction temperature turned out to be 30^o^C. Similar high enzymatic activity was observed at 20^o^C, which accounted for 79% of the activity at 30^o^C. Lower temperature of 10^o^C reduced the enzyme activity to approximately 1/3 of the activity at 30^o^C. Similar activity was observed for increased temperature at 40^o^C, whereas further increasing temperature to 50^o^C resulted in a complete loss of activity (Fig. 1C).

### Acyl-CoA and diacylglycerol preferences of *Pt*DGAT2b

Derived optimization data were applied in assays determining substrate preference of *Pt*DGAT2b. In these assays, two different molecular species of diacylglycerol were used as an acyl acceptor: di-16:0-DAG and di-18:1-DAG. Ten different [^14^C]-acyl-CoAs were used as an acyl donor. In case of testing substrate preference toward 16:1 and long-chain-polyunsaturated acyl donors, unlabeled acyl-CoAs and [^14^C]-labeled di-18:1-DAG were used. All reactions were conducted for 30 min at 30^o^C with continuous shaking (1250 rpm).

**Fig. 2:**
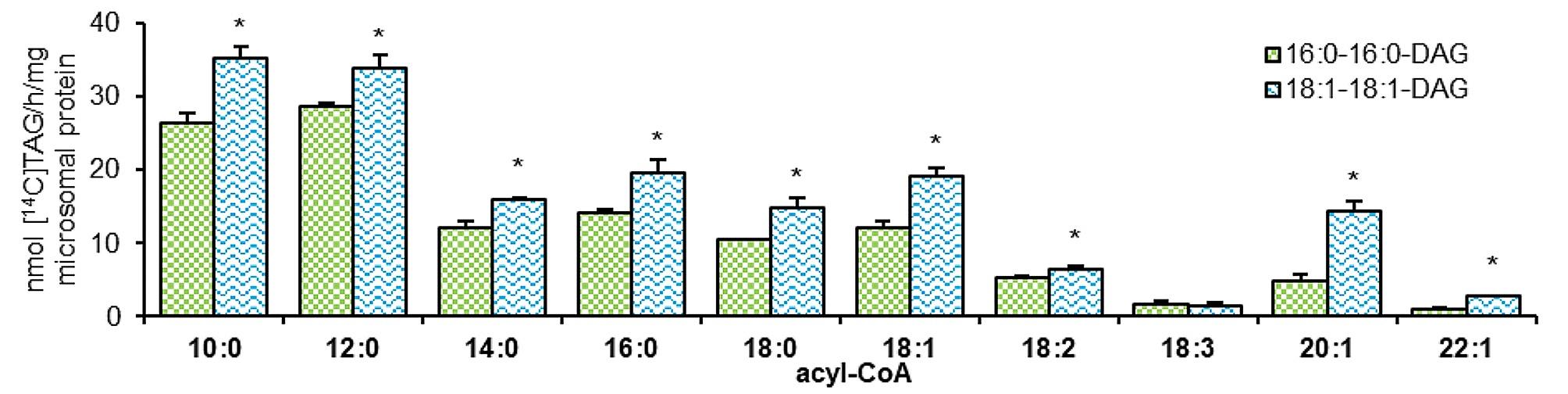
Acyl-CoA and diacylglycerol specificities of *Pt*DGAT2b. Chart represents substrate specificity with ten different [^14^C]acyl-CoAs and two diacylglycerols: di-16:0-DAG and di-18:1-DAG. Error bars present standard deviations between independent biological replicates. Asterisks above bars denote statistical significance of the *Pt*DGAT2b activity toward the same acyl donor and different tested acceptors. Statistical significance was calculated in two-tailed Student’s t-test: * – *p* ≤ 0.05

In assays, in which radiolabeled acyl-CoA and unlabeled DAGs were used, *Pt*DGAT2b utilized both acyl acceptors, however di-18:1-DAG was favored. The activity varied depending on the used acyl-CoAs. The biggest differences were observed for 20:1-CoA and 22:1-CoA, which were three times better utilized with di-18:1-DAG than with di-16:0-DAG. In assays with di-18:1-DAG, the 10:0-CoA, 14:0-CoA, 18:0-CoA, 18:1-CoA (18:1^∆9^; oleic acid) and 18:2-CoA (18:2^∆9,12^; LA; linoleic acid) were approximately 24-37% better incorporated. The slightest difference was noticed for reactions with 12:0-CoA.When di-18:1-DAG was used, its utilization was only 15% higher than with di-16:0-DAG. The only exception was 18:3-CoA (18:3^∆9,12,15^; ALA; α-linolenic acid). When it was used as an acyl donor, there was no statistically significant difference in the incorporation between adding di-16:0-DAG and di-18:1-DAG as an acceptor. (Fig. 2).

The activity of *Pt*DGAT2b in the presence of di-16:0-DAG was the highest toward 12:0-CoA and accounted for about 28.8 nmol of [^14^C]TAG synthesized per 1 h by 1 mg of microsomal protein. Similarly, 10:0-CoA was used with activity equal to 26.4 nmol [^14^C]TAG/h/mg microsomal protein. The following acyl-CoAs were also well-utilized by the enzyme, in order of decreasing efficiency: 16:0-, 14:0-, 18:1- and 18:0-CoA. *Pt*DGAT2b activity with these acyl-CoAs ranged from 10.4 to 14.2 nmol [^14^C]TAG/h/mg microsomal protein. The least preferred acyl-CoAs were 18:2-, 20:1-CoA and negligible activity was detected toward 18:3- and 22:1-CoA (Fig. 2).

The acyl-CoA preference of *Pt*DGAT2b, with unlabeled di-18:1-DAG, was quite similar to di-16:0-DAG. The highest activity was detected for 10:0-CoA which amounted to 35.2 nmol [^14^C]TAG/h/mg microsomal protein and for 12:0-CoA equal to 33.9 nmol [^14^C]TAG/h/mg microsomal protein. Next, well utilized donors were 16:0-, 18:1-,14:0-, 18:0-, and 20:1-CoA, toward which activity accounted for from 19.6 to 14.3 nmol [^14^C]TAG/h/mg microsomal protein. The worst accepted acyl-CoAs were both 18 C polyunsaturated acyl-CoA and 22:1-CoA (Fig. 2).

**Fig. 3:**
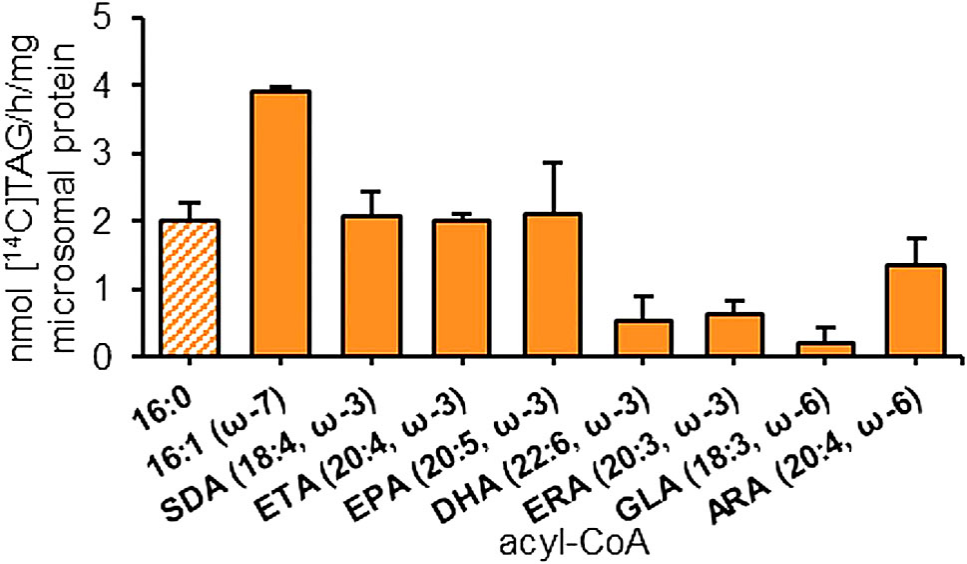
Acyl-CoA specificities of *Pt*DGAT2b toward 16C fatty acids and LC-PUFAs conjugated with coenzyme A. Chart represents substrate specificity toward seven different polyunsaturated acyl-CoAs belonging to ω-3 and ω-6 pathways and two 16 C acyl-CoAs. As an acceptor, radiolabeled diacylglycerol – [^14^C]di-18:1-DAG was used. Error bars present standard deviations between independent biological replicates

Since tested *Pt*DGAT2b is native to a marine organism producing LC-PUFAs, we verified its substrate preference toward these acyl-CoA donors. Additionally, we tested *Pt*DGAT2b preference toward 16:1-CoA (ω-7), as palmitoleic acid is present in abundance in *P. tricorunutm* [9]. In this assay variant the [^14^C]labeled di-18:1-DAG and unlabeled acyl donors were used. 16:0-CoA was assigned as a reference since it was used in both assays with and without a [^14^C]-labeled acyl acceptor. Considering the results of both tests, the activity of *Pt*DGAT2b towards 16:0-CoA was approximately 9.8-fold lower in assays with labeled DAG compared to the assays with unlabeled DAG. This result indicated very efficient utilization of endogenous DAG pool (Figs. 2 and 3). Nevertheless, comparison of the activities toward 16:0-CoA in both assays allowed for estimated determination of the preferences toward all tested acyl-CoAs (Figure S3).

The activity of *Pt*DGAT2b toward 16:1-CoA was the highest and accounted for about 3.9 nmol [^14^C]TAG/h/mg microsomal protein. It was also the best utilized acyl donor among all tested. It constituted 196% of the activity toward 16:0-CoA (Fig. 3). At the level comparable to activity toward 16:0-CoA, very efficient utilization in TAG formation by the enzyme was detected for SDA (18:4^∆6,9,12,15^; stearidonic acid), ETA (20:4^∆8,11,14,17;^ eicosatetraenoic acid) and EPA acyl-CoA. These fatty acids belong to ω3-pathway of LC-PUFAs biosynthesis. The preference toward DHA-CoA was about four times lower than the other ω3-fatty acids. The utilization of 20:4-CoA (20:4^∆5,8,11,14^; ARA; arachidonic acid; ω6) and 18:3-CoA (18:3^∆6,9,12^; GLA; γ-linolenic acid; ω6) was about 31% and 83%, respectively lower than the utilization of 16:0-CoA. Activity toward 20:3-CoA (20:3^Δ11,14,17^; ERA eicosatrienoic acid; ω6), belonging to the intermediates of an alternative ∆^8^-pathway, was comparable to DHA-CoA utilization (Fig. 3; Figure S3).

### The effect of *PtD*GAT2b transient expression on lipid composition of *Nicotiana benthamiana* leaves

To verify the effect of *Pt*DGAT2b action *in planta*, we conducted transient expression experiments on *Nicotiana benthamiana* leaves. Gene encoding *Pt*DGAT2b was expressed with two other genes encoding p19 – the silencing suppressor and GFP - the reporter gene, both of which were also introduced into leaves as a control trial, without the gene of interest.

**Fig. 4:**
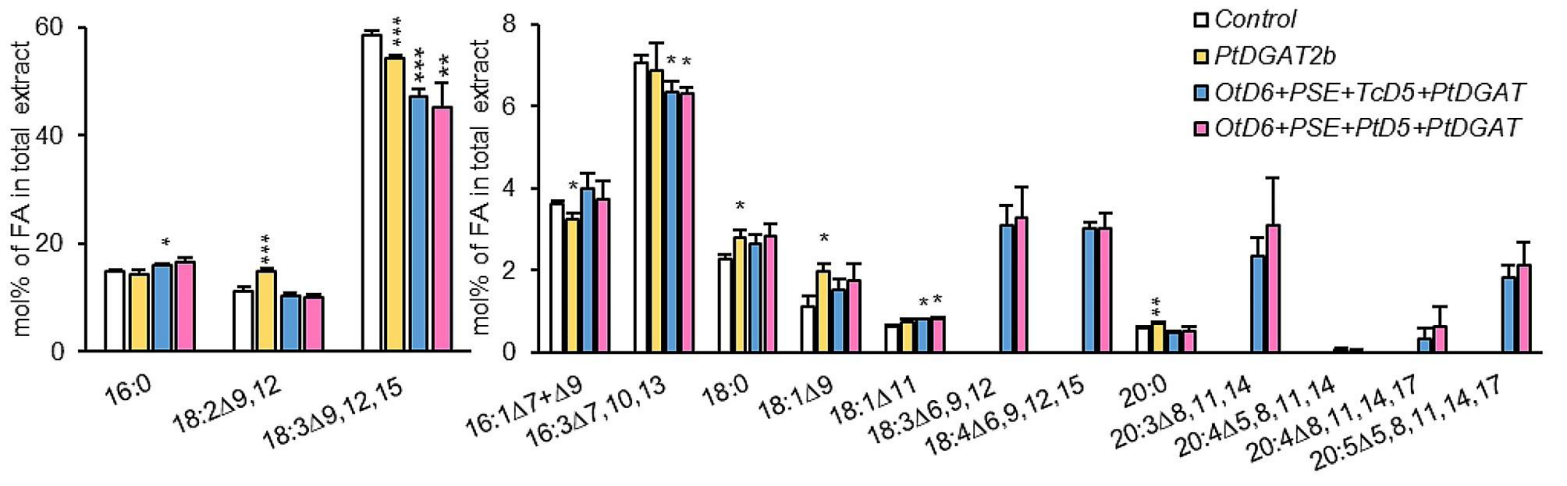
Fatty acid composition in acyl-lipids of *Nicotiana benthamiana* leaves obtained through agroinfiltration with *Pt*DGAT2b encoding gene and gene combinations aimed at producing EPA (20:5^∆5,8,11,14,17^). The results show the effects of control (p19 + GFP), p19 + GFP + *Pt*DGAT2b and co-expression of p19 + GFP + *Pt*DGAT2b with gene combinations encoding: *Ot*D6 + PSE + *Tc*D5 and *Ot*D6 + PSE + *Pt*D5. Error bars present standard deviations between independent biological replicates. Asterisks above bars denote statistical significance compared to control. Statistical significance was calculated in two-tailed Student’s t-test: * – *p* ≤ 0.05; ** - *p* ≤ 0.01; *** - *p* ≤ 0.001

Analysis of fatty acid composition in total lipid extract showed that expression of *Pt*DGAT2b in tobacco leaves strongly affects 18:2 and 18:3 composition (Fig. 4). The content of 18:2 increased by 3.8% (to 15% of all fatty acids) and the content of 18:3 decreased by 4.3% (to 54.3% of all fatty acids) compared to the control. Reduced content was also noticed for 16:1, whereas no change was detected for 16:0. Relative content of other fatty acids were slightly elevated by approximately 0.1–0.9% over their relative content in the control.

**Fig. 5:**
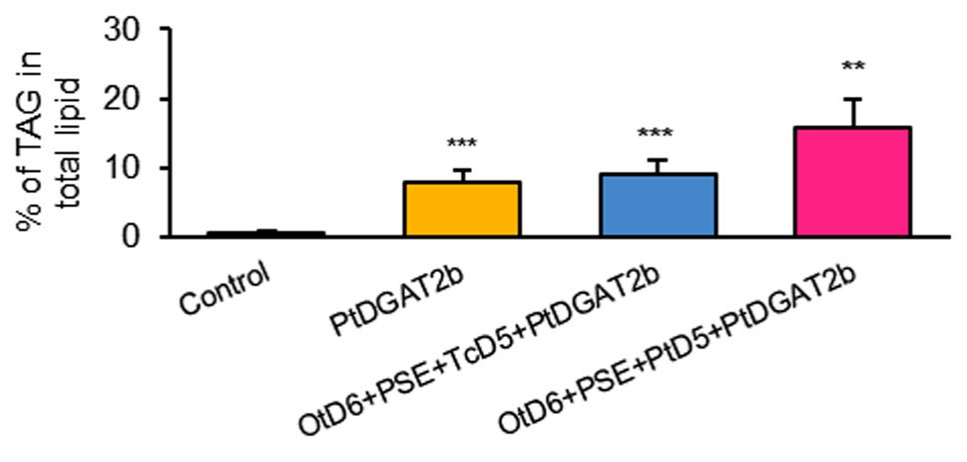
Changes in the content of triacylglycerols in *Nicotiana benthamiana* leaves caused by introduced *Pt*DGAT2b action and its action together with co-introduced *Ot*D6 + PSE + *Tc*D5 and *Ot*D6 + PSE + *Pt*D5 enzyme combinations aimed at producing EPA (20:5^∆5,8,11,14,17^). Asterisks denote statistical significance compared to control. Statistical significance was calculated in two-tailed Student’s t-test: * – *p* ≤ 0.05; ** - *p* ≤ 0.01; *** - *p* ≤ 0.001; a - *p* ≤ 0.05

**Fig. 6:**
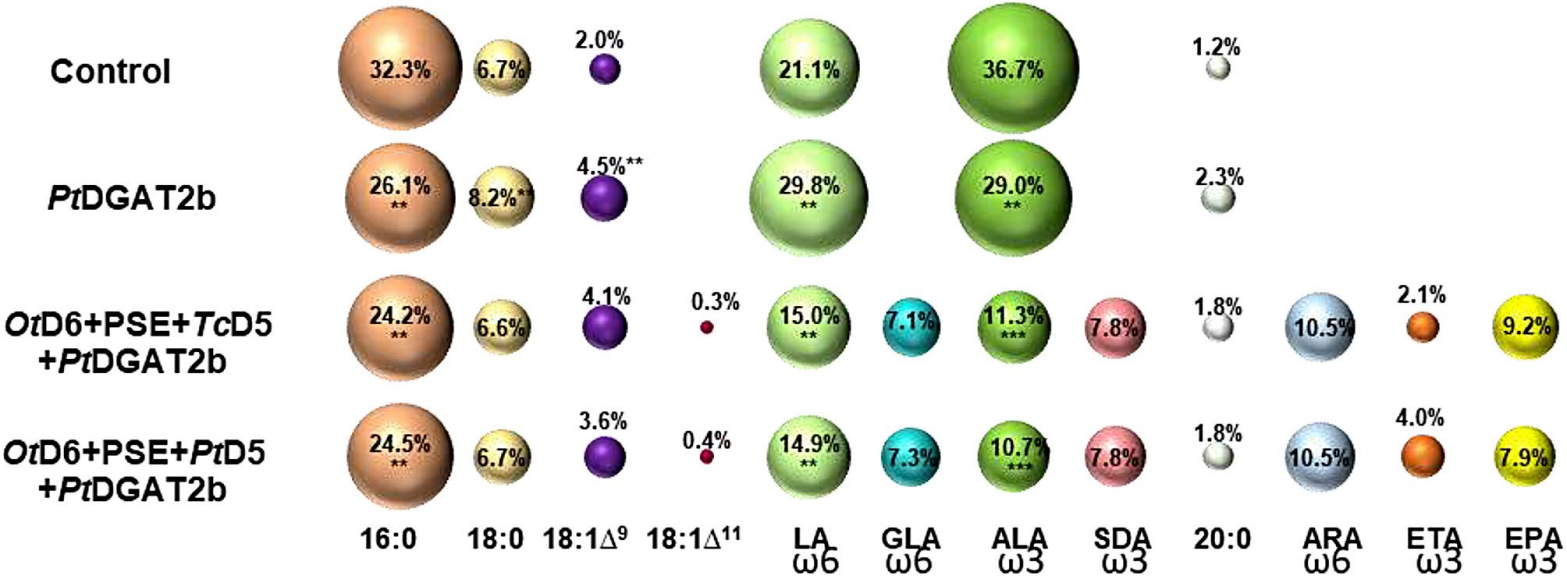
Fatty acid composition in triacylglycerol pool of *Nicotiana benthamiana* leaves derived through agroinfiltration with *Pt*DGAT2b encoding gene separately and together with gene combinations aimed at producing EPA (20:5^∆5,8,11,14,17^). The results concern TAG of control leaves (p19 + GFP), TAG of leaves with p19 + GFP + *Pt*DGAT2b and TAG of leaves with introduced p19 + GFP + *Pt*DGAT2b together with co-introduced *Ot*D6 + PSE + *Tc*D5 or *Ot*D6 + PSE + *Pt*D5 enzyme combinations. The size of the spheres correlates with the percentage of each fatty acid in the whole pool. Asterisks denote statistical significance compared to control. Statistical significance was calculated in two-tailed Student’s t-test: * – *p* ≤ 0.05; ** - *p* ≤ 0.01; *** - *p* ≤ 0.001; a - *p* ≤ 0.05. Standard deviations are presented in Table S1

Since the DGAT enzyme is responsible for the triacylglycerol biosynthesis, we also determined the changes in the TAG pool. We observed an increased production of TAG, with relative amount increasing from 0.7% (detected for control) to 8.0% of all acyl-lipids (Fig. 5). Observed fatty acid composition tendency in overall acyl-lipid pool was also noticeable in fatty acid composition of the TAG pool. The relative amount of 18:3 increased to 29%, hence by 7.7% compared to control, whereas for 18:2 reduction by 7.7% (to 29% of all fatty acids) was determined. Likewise, reduction of the relative amount was noticed 18:0, 18:1, 20:0 and oscillated between 1.1 and 2.5%. Unlike the fatty acid composition of total acyl-lipids, in TAG pool the content of 16:0 declined by 6.2%, reaching 26.1% of all fatty acids in this pool (Fig. 6). Described differences were only observed in the TAG pool, as they were not detected in the pool of other acyl-lipids all together (Table S2).

### *PtD*GAT2b-mediated EPA accumulation *in planta*

*Pt*DGAT2b was also used in experiment aimed at EPA biosynthesis in *N. benthamiana* leaves to determine its potential in further stable oilseed plant transformation. *Pt*DGAT2b was co-expressed with two gene combinations. One combination consisted of ∆^6^-desaturase from *Osterococcus tauri*, ∆^6^-elongase from *Physcomitrium patens* and ∆^5^-desaturase from *Thraustochytrium sp*. The second combination differed by ∆^5^-desaturase, which was cloned from *P. tricornutum*. Both combinations have been already tested as they efficiently synthesized LC-PUFAs (Table S3; [3, 28]).

Co-expression of both gene combinations with *Pt*DGAT2b resulted in significant changes in fatty acid composition in acyl-lipids. Newly synthesized ω-3 and ω-6 fatty acids constituted up to 10.7% and 12.2% of all fatty acids, respectively for *Pt*D6 + PSE + *Tc*D5 + *Pt*DGAT2b and *Pt*D6 + PSE + *Pt*D5 + *Pt*DGAT2b. The relative amount of two fatty acids, which are the intermediate products of ω6- and ω3-pathways, respectively: GLA and SDA, oscillated between 3.0 and 3.3% of all fatty acid content. Subsequent fatty acid, produced in ω6-pathway via elongation process – DGLA (20:3^∆8,11,14^; dihomo-γ-linolenic acid, ω6) also constituted 2.4% and 3.1% of all fatty acids for combinations *Pt*D6 + PSE + *Tc*D5 + *Pt*DGAT2b and *Pt*D6 + PSE + *Pt*D5 + *Pt*DGAT2b, respectively. Further ∆^5^-desaturation, resulted in a negligible relative content of ARA. In parallel, other fatty acids belonging to ω3-pathway were synthesized. The content of ETA (20:4^∆8,11,14,17^; eicosatetraenoic acid) accounted for 0.3% and 0.6% of all fatty acids in acyl-lipids, respectively for *Pt*D6 + PSE + *Tc*D5 + *Pt*DGAT2b and *Pt*D6 + PSE + *Pt*D5 + *Pt*DGAT2b. Correspondingly for these combinations EPA content equal to 1.8% and 2.1% was detected, which corresponds to 16.8% and 17.2% of all exogenous fatty acids synthesized. Such a significant production of non-native fatty acids affected the composition of endogenous fatty acids in tobacco leaves. The relative content of 18:3 decreased by 11.4% and 13.3% from 58.6% detected for control, accordingly for *Pt*D6 + PSE + *Tc*D5 + *Pt*DGAT2b and *Pt*D6 + PSE + *Pt*D5 + *Pt*DGAT2b. For 18:2 no statistically significant changes were reported, although single expression of *Pt*DGAT2b increased its level, which might indicate preferential use of 18:2 as a substrate by ∆^6^-desaturase (Fig. 4).

Since triacylglycerol is a major reservoir of lipids in oilseed plants, we determined the content and composition of this lipid class. Both tested combinations resulted in elevated level of the TAG pool, which accounted for 9% and 15% of all acyl-lipids, respectively for *Pt*D6 + PSE + *Tc*D5 + *Pt*DGAT2b and *Pt*D6 + PSE + *Pt*D5 + *Pt*DGAT2b. Boosted TAG content was also observed for combinations without PtDGAT2b co-expression; however, they were still significantly lower compared to co-expression with *Pt*DGAT2b (Fig. 5; Table S3). The TAG pool composition of newly synthesized ω-3 and ω-6 fatty acids derived via *Pt*D6 + PSE + *Tc*D5 + *Pt*DGAT2b and *Pt*D6 + PSE + *Pt*D5 + *Pt*DGAT2b actions amounted to 36.7% and 37.5%, respectively, among which EPA consisted of 9.2% and 7.9% (statistically significantly compared to action of gene combination without *Pt*DGAT2b; Table S3). Non-native fatty acids belonging to ω3-pathway constituted 52% of all exogenous fatty acids for both combinations. The rest constituted of ω6 fatty acids, among which ARA content was the highest (10.5% of all detected fatty acids in the TAG pool). Similar to fatty acid composition of total acyl-lipids, declined content of linolenic acid was also noticed in the TAG pool. Its relative amount reduced from about one-third of content presented in control to 11%. The content of palmitic and linoleic acid decreased significantly by on average 8% for 16:0 and by 6% for 18:2 (Fig. 6). DGLA was not detected in the TAG pool, as it was probably incorporated into other lipid classes (Table S2).

## Discussion

The mechanism of TAG biosynthesis is inextricably linked to the enzymes involved in the last step of their synthesis – DGAT and PDAT. The activity of these enzymes is not only a limiting factor of TAG biosynthesis, but also influences composition of the produced storage lipids [29, 30]. Based on a study done on Arabidopsis mutant lines of *dgat1* and *pdat1* has shown that both enzymes play complementary role, while their double knockout is lethal [31]. Knockout of *dgat2* did not show any significant changes in oil content compered to wildtype. Nevertheless, many other studies point to the significance of DGAT2 enzymes since they can exhibit high specificity toward unusual fatty acids such as eleostearic acid, ricinoleic acid, erucic acid, and vernolic acid in plants synthesizing of these fatty acids [32–34]. In such plants, DGAT2 seed expression is much higher throughout seed development than in crops producing common fatty acids [35].

Oleaginous microalgae accumulate TAGs in high quantity in response to various abiotic stresses [23, 36]. In addition, oleaginous microalgae are a primary source of LC-PUFAs. Therefore, they are a great source of enzymes and a model for studying LC-PUFAs biosynthesis. One of the model diatoms – *P. tricornutum* can accumulate large amounts of TAGs – up to 75% of total lipids under nitrogen-deficient conditions. EPA is the dominant fatty acid enriched in galactolipids whereas it is incorporated with a low amount in the composition of these TAGs [37]. In this study, we decided to investigate the possible role of *P. tricornutum* DGAT in the increased accumulation of LC-PUFAs in plant TAG. Our study focused on *Pt*DGAT2b, which ability to produce TAG has been already confirmed by Gong et al. [19]. That study also indicated that *Pt*DGAT2 is not strongly regulated by nitrogen deficiency unlike *Pt*DGAT1, which suggests a crucial role in storage lipid accumulation in nitrogen-replete conditions. That feature was attributed primarily to the *Pt*DGAT2b, on which we focused our attention.

Till now many DGAT enzymes native to microalgae have been identified, but only a few have been thoroughly characterized biochemically. Prior to determination of the enzyme’s substrate specificity, we evaluated the best parameters for in vitro enzymatic reaction carried by *Pt*DGAT2b, expressed in baker’s yeast. As our assay showed, optimization of microsomal fraction concentration can significantly affect the activity, which is essential for proper evaluation of enzyme’s biochemical features. Currently, many studies on the DGAT activity use different concentrations, which have not been confirmed by optimization assays. Furthermore, even though our obtained values of the other two optimized parameters, reaction time and temperature overlapped with other assays conducted for plant and algae DGAT activity assessment in yeast, only a minor part of other studies include optimization of these parameters [34, 38–40]. For *Pt*DGAT2b, detected maximum activity at 30^o^C, corresponding to optimal baker’s yeast growth temperature, is not consistent with *P*. *tricorunutum* culturing temperature, which is 19^o^C [8, 41]. Nevertheless, this enzyme also maintained a quite high activity at 20^o^C reaching 79% of maximum activity at 30^o^C. The disparity between the native temperature environment of an enzyme and its biochemical properties is a common characteristic among acyltransferases [33, 42, 43]. We cannot exclude, that observed discrepancies result from membrane composition differences between native membrane of diatom and yeast, in which their activity was tested. Nevertheless, evidence indicating ability of *Pt*DGAT2b to operate TAG biosynthesis in not only optimal growth temperature conditions but also in temperature stress conditions can be promising in engineering of *P. tricornutum* toward boosted storage lipid production or in plant lipid metabolism engineering.

To verify substrate specificity of *Pt*DGAT2b we used two acyl acceptors: di-16:0-DAG and di-18:1-DAG, in in vitro assays. The first one was chosen based on the diacylglycerol molecular species naturally occurring in *P. tricornutum*, which are abundant in 16:0 and 16:1 [44]. In plant, such DAG molecules are present in lower quantity, since 18C unsaturated fatty acids are the most abundant in most plant DAG pools [45, 46]. For this reason di-18:1-DAG was chosen as the second tested acyl acceptor. Despite the above-mentioned differences in plant and diatom DAG species, *Pt*DGAT2b favored di-18:1-DAG. The ability to utilize this acceptor in relation to di-16:0-DAG depended on the used acyl-CoAs and ranged from 115% to even 166%, with the exception of 18:3-CoA, which was better utilized with di-16:0-DAG. One of the highest activities with both acyl acceptors was noted for medium-chain acyl-CoAs (10:0-CoA and 12:0-CoA), although these compounds were not detected in *P. tricornutum*. In assays with di-18:1-DAG the top preference was observed toward 16:1 (ω-7), one of the native fatty acids for diatoms. Both findings make *Pt*DGAT2b a promising enzyme for crop modification, for enriching said crops in lipids with medium-chain or ω-7 fatty acids, useful in biofuels and industrial applications. Nevertheless, our main goal was to verify whether these enzymes accept LC-PUFAs and whether it could be used successfully *in planta* LC-PUFAs biosynthesis pathway construction. The utilization of three ω-3 fatty acids – SDA, ETA and EPA were third in order of the best ones conducted by *Pt*DGAT2b, on par with 16:0 and 18:1 utilization. DHA-CoA was much less accepted, which can be attributed to poor DHA distribution in neutral lipids in *P. tricornutum* [9]. The *Pt*DGAT2b preference toward ω-6 LC-PUFAs was significantly lower, which might be a key feature of *Pt*DGAT2b for its native diatom’s metabolism, as the diatom is able to accumulate high amounts of EPA, while it accumulates other intermediates in trace level. Our results are consistent with the study done by Zhang et al. [23], where *Pt*DGAT2b’s high preference for 16:0-, 16:1- and 20:5-CoA was observed in the presence of di-16:1-DAG, the most abundant DAG species in *P. tricornutum*, however present in small quantities in plant.

This observation led us to further investigation of the potential of *Pt*DGAT2b in ω-3 LC-PUFA accumulation *in planta*. Firstly, we verified the effect of separate expression of *Pt*DGAT2b on acyl-lipid composition of *N. benthamiana* leaves. The activity of this enzyme resulted in elevated levels of stearic, oleic and linoleic acids both in total acyl-lipids and in the TAG pool, at the expense of the relative amounts of palmitic and linolenic acid in the TAG pool. While the accumulation patterns of 18:0, 18:1 and 18:3 are consistent with the substrate preference of *Pt*DGAT2b, no such relationship was observed for 16:0 and 18:2. Nevertheless, such differences can be caused by the availability of acyl-CoA species and/or competition for substrates with other enzymes. Moreover, this phenomenon can be plant or even organ/tissue specific. When *Pt*DGAT2b was co-expressed with gene combinations aimed at EPA production, the relative amount of linoleic acid significantly declined, just as the relative amount of linolenic acid, which was caused by the effective activity of ∆^6^-desaturase. The expression of this desaturase led to γ-linolenic acid or stearidonic acid biosynthesis, respectively from 18:2 and 18:3. Final product of ω-3 LC-PUFA biosynthesis pathway reached 9.2% and 7.9% of all fatty acids in the TAG pool, respectively for gene combinations: *Ot*D6 + PSE + *Tc*D5 and *Ot*D6 + PSE + *Pt*D5 co-expressed with *Pt*DGAT2b. These relative amounts correspondingly constitute 25.1% and 21.1% of synthesized non-native fatty acids accumulated in the TAG pool. This makes *Pt*DGAT2b an excellent enzyme with great further potential to increase the accumulation of ω-3 LC-PUFA in oilseeds, as the TAG pool is their main site of fatty acid accumulation. Nevertheless, it should be noted that accumulation of arachidonic acid was the highest out of the exogenous fatty acids in the TAG pool, albeit its relative amount did not stand out in the total acyl-lipid composition. The presence of fatty acids such as GLA or ARA in this storage lipid pool is surprising because *Pt*DGAT2b did not exhibit a preference for them (activity with GLA constituted only 16% of activity with SDA and ARA constituted 64% of activity with EPA). This could have been caused by the action of endogenous TAG-synthesizing enzymes, which substrate preference is unknown. In future, to further stimulate the EPA biosynthesis in the TAG pool, additional enzymes catalyzing the conversion of ARA to EPA, the ω-3 desaturase with ability to incorporate a double bond at ∆^17^ site, should be introduced. Increased content of the TAG pool should also be mentioned in relation to our results from this study. In all introduced gene combinations, expression of *Pt*DGAT2b separately and its co-expression with *Ot*D6 + PSE + *Pt*D5 combinations aimed at EPA production significantly elevated TAG content from 0.7% in control to 15.7% of all acyl-lipids.

## Conclusions

Our data present novel and relevant evidence that *Pt*DGAT2b is a promising enzyme that can be used in *in planta* metabolic engineering directed not only to boost TAG content but also to accumulate significant quantities of LC-PUFAs, as well as in research dedicated to increasing the amount of medium-chain and ω-7 fatty acids in plant storage lipids.

## Experimental procedures

### Chemicals

Acyl-CoAs ([1-^14^ C]-labeled an unlabeled) used for enzymatic assays were synthesized according to the method developed by Sánchez et al. [47]. [1-^14^C]-fatty acids and triolein [carboxyl-^14^C] were purchased from Perkin Elmer or American Radiochemicals. Labeled diacylglycerol was derived through partial lipase treatment of triolein [carboxyl-^14^C] with *Rhizopus arrhizus* lipase (Sigma-Aldrich). The lipase products were separated on TLC plates (silica gel 60; Sigma-Aldrich). The DAG was eluted from the silica with methanol: chloroform (2:1, v/v), extracted with chloroform by Bligh and Dyer method (1959). Non-radioactive fatty acids and diacylglycerol were ordered from Larodan or Sigma-Aldrich. Internal standard of heptadecanoic acid methyl ester, coenzyme A and TAG standard for thin-layer chromatography were purchased also from Sigma-Aldrich.

### Vector construction and yeast transformation

Gene encoding DGAT2b from *Phaeodactylum tricornutum* was identified by Gong et al. [19] and was assigned to the accession number JQ837823. Tested gene was ordered as a synthetic codon optimized for expression in *Saccharomyces cerevisiae* (ThermoFisher Scientific) and firstly cloned by Gateway cloning into pDONR221 plasmids and then into pYES-derived plasmid pDEST, under control of the GAL1 promoter. Whole procedure was done according to manufacturer’s instruction (Invitrogen).

The designed construct of pDEST52-GAL1::*Pt*DGAT2b was introduced in to TAG-deficient *S. cerevisiae* strain H1246 (MATa are1-D::HIS3, are2-D::LEU2, dga1-D::KanMX4, lro1-D::TRP1 ADE2; [27]).The transformation yeast protocol was done according to the modified LiAc/SS carrier DNA/PEG method [48]. Recombinant yeast cells were selected in synthetic uracil dropout medium supplemented with 2% of glucose.

### Yeast microsomal preparation and assay of *Pt*DGAT2b

The transformed yeast cells harboring empty plasmid pYES-DEST52 or plasmid pDEST52-GAL1::*Pt*DGAT2b were cultivated for 48 h at 30^o^C in synthetic uracil dropout medium supplemented with 2% of galactose to induce gene expression and protein synthesis. After that time, yeast cultures were harvested via centrifugation (10 min, 1500x*g*, 4^o^C), washed twice with distilled water followed by centrifugation again. Obtained pellets were resuspended in buffer containing 20mM Tris-HCl (pH 7.9),10 mM MgCl_2_; 1 mM EDTA; 5% glycerol, 0.3 M ammonium sulphate and protease inhibitor and transferred into tubes containing glass beads (0.45–0.5 mm in diameter). Tubes with yeast resuspension were placed in Mini Bead Beater-8 and shaken 10-times for 30 s with 5-minutes break after five times. Disrupted yeasts were centrifuged (10 min, 1500xg, 4^o^C) and obtained supernatants were subjected to ultracentrifugation at 100000x*g* for 1 h 30 min at 4^o^C. Resulting pellets were resuspended in 0.1 M potassium buffer (pH 7.2) and stored at -80^o^C. Aliquots of microsomal fraction containing appropriate amount of microsomal protein were further used in enzyme assays.

All enzymatic reactions were conducted according to the methods described by Jeppson et al. [40], in which DAG species, added to reaction mixtures, were dissolved in dimethyl sulphoxide (DMSO). Experiments aimed at optimization of the best parameters of the in vitro assays of tested enzyme were performed using [^14^C]16:0-CoA and di-18:1-DAG. The determined optimal parameters subsequently of amount of microsomal fraction, reaction time and reaction temperature, were applied successively in conducted assays. Detailed study of substrate specificity was performed in two variants. The first one, in which non-radioactive diacylglycerol either 10 nmol of di-16:0-DAG or di-18:1-DAG was used with 10 nmol of ten different [^14^C]-labeled acyl-CoAs, added separately: decanoyl-CoA ([^14^C]10:0-CoA), lauroyl-CoA ([^14^C]12:0-CoA), myristoyl-CoA ([^14^C]14:0-CoA), palmitoyl-CoA ([^14^C]16:0-CoA), stearoyl-CoA ([^14^C]18:0-CoA), oleoyl-CoA ([^14^C]18:1-CoA), linoleoyl-CoA ([^14^C]18:2-CoA), linolenoyl-CoA ([^14^C]18:3-CoA), eicosenoyl-CoA ([^14^C]20:1-CoA) and erucoyl-CoA ([^14^C]22:1-CoA). In the second variant 10 nmol of [^14^C]-di-18:1-DAG was used and 10 nmol non-radioactive acyl-CoA: palmitoyl-CoA (16:0-CoA), palmitoleoyl-CoA (16:1^∆9^-CoA), γ-linolenoyl-CoA (18:3^∆6,9,12^-CoA), eicosatetraenoyl-CoA (18:4^∆6,9,12,15^-CoA), eicosatrienoyl-CoA (20:3^∆11,14,17^-CoA), arachidonoyl-CoA, (20:4^∆5,8,11,14−^CoA), eicosatetraenoyl-CoA (20:4^∆8,11,14,17^-CoA), eicosapentaenoyl-CoA, (20:5∆^5,8,11,14,17^-CoA), docosapentaenoyl-CoA (22:6^∆4,7,10,13,16,19^-CoA). Each assay was carried out in the final volume of 100 µl in buffer containing 0.05 M HEPES (pH 7.2), 5 mM MgCl_2_, 100 ug of BSA and 21 µg of yeast microsomal protein. Reaction was conducted 30 min at 30^o^C with continuous shaking 1250 rpm. After the incubation, lipid fractions were extracted into chloroform by Bligh and Dyer method [49]. Chloroform fractions were separated on TLC plates (silica gel, 60; SigmaAldrich) in hexane: diethyl ether: acetic acid (70:30:1; v:v:v). [^14^C]TAG products were visualized and measured by electronic autoradiography (Instant Imager, Packard).

### Plant expression vector and *Nicotiana benthamiana* infiltration

For construction of plant expression vectors carrying desired genes, the Golden Gate cloning method was used [50]. Gene encoding DGAT2b from *Phaeodactylum tricornutum* and set of genes aimed at EPA production: ∆^6^-acyl-CoA desaturase from *Osterococcus tauri*, ∆^6^-elongase from *Physcomitrium patens*, ∆^5^-lipid-linked desaturase from *Phaeodactylum tricornutum* and *Thraustochytrium sp.* were ordered as a synthetic, codon optimized sequence (ThermoFisher Scientific). All of them were introduced into acceptor vector (from level 1) under the enhanced Cauliflower mosaic virus 35 S promotor and octopine synthase terminator. Genes encoding p19 and GFP were introduced into pXZP393 vector via Gateway cloning method.

Constructed plant expression vector was used in electroporation of *Agrobacterium tumefaciens* GV3101 to obtain separate lines carrying individual vectors. Agrobacterium colonies were used for *Nicotiana benthamiana* infiltration (*N. benthamiana* provided from sources of Swedish University of Agricultural Science in Alnarp, Sweden). Overnight liquid cultures of Agrobacterium were supplemented with acetosyringone to a final concentration 100 µM and grown for an additional 3 h. After incubation, the cultures were spun down (3000xg, 5 min) and resuspended in Infiltration Media (pH 5.7) containing 5 mM MgCl_2_, 5 mM MES and 100 µM acetosyringone. Based on the measured optical density, aliquots equal to OD 0.2 of different Agrobacterium culture carrying desired gene were mixed and refilled to 30 ml with Infiltration Media.

*Nicotiana benthamiana* plant used for Agroinfiltration was 4–5 weeks old. The mixtures containing appropriate gene combination were pressed to the underside of the leaf by 1 ml syringe. Both before and after the treatment, *N. benthamiana* was cultivated at day/night conditions, which lasted for 16 h/8 h and relative humidity set to 60%. The temperature was set to 23 °C/20°C for 13 h/11 h with the light (260 µmol/m^2^/s µmol photosynthetic photon flux density).

### Lipids analysis

Five days after treatment, infiltrated leaf areas were visualized by GFP marker. Detected, fluorescent areas were excised and freeze-dried for 3 days, after which dry mass weight was determined. Homogenization of freeze-dried leaves was conducted according to the method described by Bligh&Dyer [49]. The chloroform fractions obtained after extraction were dried under N_2_ and dissolved in a known volume of chloroform. The chloroform volume corresponding to 10 mg of dry leaf weight was used for acyl-lipid assessment, whereas volume corresponding to 50 mg was used for TLC method to separate triacylglycerol pools and for determination of their composition.

To separate TAGs, aliquots of chloroform fractions were concentrated and applied to TLC plates (silica gel 60), which after complete drying were placed in TLC chamber filled with mobile phase containing mixture of heptane: diethyl ether: acetic acid (70:30:1; v: v:v). After lipid separation, dried TLC plates were sprayed with primuline and lipid classes were visualized under UV light. Silica gel areas with localized TAG were scraped off and supplied with 2 ml of 2% H_2_SO_4_ (methylation mixture) in dry methanol and incubated for 40 min at 90^o^C to produce derivatives of methyl esters. To determine the acyl-lipid composition in total extract, aliquots of chloroform fractions were dried under N_2_ and supplied with methylation mixture and subjected to methylation process, omitting the TLC separation. After incubation, probes with the obtained fatty acids methyl esters were extracted by heptane and separated on CP-Wax 58 FFAP CB column (Agilent) in a gas chromatograph with a flame ionization detector (Agilent 8860 GC System). As an internal standard 17:0 methyl ester was added prior to the methylation step.

### Accession numbers

Nucleotide sequence data are available under the accession number: p19 - P69516.1; GFP - ABE28520; ∆^6^ acyl-CoA desaturase from *Osterococcus tauri* - XM_003082530.1; ∆^6^ elongase from *Physcomitrium patens* - AF428243.1; ∆^5^ lipid-linked desaturase from *Phaeodactylum tricornutum -* GQ352540.1 and *Thraustochytrium sp. -* AF489588; acyl-CoA: diacylglycerol acyltransferase 2b from *Phaeodactylum tricornutum* - JQ837823.

### Electronic supplementary material

Below is the link to the electronic supplementary material.

Supplementary Material 1

## Data Availability

The datasets used and/or analyzed during the current study will be available from the corresponding author on reasonable request.

## Decarations

### Statement related to used plant material

Formal identification of the plant material used in your study was done by Sylwia Klińska-Bąchor and Kamil Demski. Research using *Nicotiana benthamiana* was carried out at Swedish University of Agricultural Science in Alnarp, Sweden, which has permission to cultivate and conduct research using this plant and where the voucher specimen is deposited.
