# Supplementary material for: Biochemical characterization of acyl-CoA:diacylglycerol acyltransferase2 from the diatom Phaeodactylum tricornutum and its potential effect on LC-PUFAs biosynthesis in planta
Source: BMC Plant Biol. 2024 Apr 23;24:309. doi: 10.1186/s12870-024-05014-7 (PMC11036593; doi:10.1186/s12870-024-05014-7)
Supplement: Supplementary file 1 — Supplementary Material 1 [file 12870_2024_5014_MOESM1_ESM.pdf]

Supplemental Data

Biochemical characterization of acyl-CoA:diacylglycerol acyltransferase2 from the diatom *Phaeodactylum tricornutum* and its potential effect on LC-PUFAs biosynthesis in planta

Sylwia Klińska-Bąchor[1,2], Kamil Demski[2], Yangmin Gong[3], Antoni Banaś[1]

[1]Intercollegiate Faculty of Biotechnology, University of Gdańsk and Medical University of Gdańsk, Gdańsk, Poland

[2]Department of Plant Breeding, Swedish University of Agricultural Sciences, Alnarp, Sweden

[3]Oil Crops Research Institute of Chinese Academy of Agricultural Sciences, Wuhan, China

[*]Correspondence author: sylwia.klinska@ug.edu.pl

Table S1 Fatty acid composition (mol%) in triacylglycerol pool of *Nicotiana benthamiana* leaves obtained through agroinfiltration with gene combinations aimed at producing EPA ($20:5^{\Delta 5,8,11,14,17}$). Data partially presented in Figure 6. Mean values and standard deviations of three independent biological replicates are presented. Asteriks denote statistical significance compared to control. Statistic values were calculated in two-tailed Student's t-test: * – p≤0.05; ** - p≤0.01; *** - p≤0.001; a - p≤0.05.

	mol% of FA in TAG pool											
	**16:0**	**18:0**	**$18:1^{\Delta 9}$**	**$18:1^{\Delta 11}$**	**$18:2^{\Delta 9,12}$**	**$18:3^{\Delta 6,9,12}$**	**$18:3^{\Delta 9,12,15}$**	**$18:4^{\Delta 6,9,12,15}$**	**20:0**	**$20:4^{\Delta 5,8,11,14}$**	**$20:4^{\Delta 8,11,14,17}$**	**$20:5^{\Delta 5,8,11,14,17}$**
**Control**	32.3 ±1.8	6.7 ±0.1	2.0 ±0.7	-	21.1 ±1.6	-	36.7 ±0.7	-	1.2 ±0.04	-	-	-
***Pt*DGAT2b**	26.1 ±0.4**	8.2 ±0.4**	4.5 ±0.5**	-	29.8 ±1.7**		29.0 ±2.3**	-	2.3 ±0.4	-	-	-
***Ot*D6+PSE+ *Tc*D5 +*Pt*DGAT2b**	24.2 ±0.6**	6.6 ±0.1	4.1 ±1.1	0.3 ±0.03	15.0 ±1.2**	7.1 ±0.8	11.3 ±1.9***	7.8 ±0.4	1.8 ±0.1	10.5 ±3.2	2.1 ±1.5	9.2 ±1.5
***Ot*D6+PSE+ *Pt*D5 +*Pt*DGAT2b**	24.5 ±0.3**	6.7 ±0.1	3.6 ±0.7	0.4 ±0.1	14.9 ±0.9**	7.3 ±0.6	10.7 ±2.2***	7.8 ±0.1	1.8 ±0.1	10.5 ±0.9	4.0 ±2.0	7.9 ±1.2

Table S2 Fatty acid composition (mol%) of lipid pool in *Nicotiana benthamiana* leaves remained after triacylglycerol separation, derived through agroinfiltration of *Pt*DGAT2b and gene combinations aimed at producing EPA (20:5$^{\Delta5,8,11,14,17}$) co-expressed with *Pt*DGAT2b. Mean values and standard deviations of three independent biological replicates are presented. Asterisks denote statistical significance compared to control calculated in two-tailed Student's t-test: * – p≤0.05; ** - p≤0.01; *** - p≤0.001; a - p≤0.05.

Lines / *Fatty acid*	Control	*Pt*DGAT2b	*Ot*D6+PSE+*Tc*D5 +*Pt*DGAT2b	*Ot*D6+PSE+*Pt*D5 +*Pt*DGAT2b
**16:0**	13.9±0.2	12.1±0.2***	13.1±0.8	13.3±0.3
**16:1$^{\Delta7+\Delta9}$**	8.3±0.3	9.2±0.9	6.8±0.6*	6.3±0.8*
**16:3$^{\Delta7,10,13}$**	7.3±0.3	8.7±0.9	8.7±0.2**	9.1±0.5**
**18:0**	2.0±0.1	1.9±0.2	1.5±0.2*	1.6±0.1*
**18:1$^{\Delta9}$**	0.9±0.3	1.2±0.5	1.0±0.2	1.1±0.2
**18:1$^{\Delta11}$**	0.5±0.03	0.6±0.03	0.6±0.04	0.6±0.02*
**18:2$^{\Delta9,12}$ [LA]**	9.8±0.8	10.7±1.9	8.0±0.3*	7.7±0.3*
**18:3$^{\Delta6,9,12}$ [GLA]**	-	-	2.5±0.4	2.6±0.5
**18:3$^{\Delta9,12,15}$ [ALA]**	57.0±1.4	55.3±0.8	52.1±1.8*	51.7±2.0*
**18:4$^{\Delta6,9,12,15}$ [SDA]**	-	-	2.6±0.1	2.6±0.3
**20:0**	0.3±0.01	0.3±0.01	0.3±0.1	0.2±0.02*
**20:3$^{\Delta8,11,14}$ [DGLA]**	-	-	1.2±0.2	1.7±0.7
**20:4$^{\Delta5,8,11,14}$ [ARA]**	-	-	-	-
**20:4$^{\Delta8,11,14,17}$ [ETA]**	-	-	0.2±0.02	0.3±0.1
**20:5$^{\Delta5,8,11,14,17}$ [EPA]**	-	-	1.1±0.1	1.3±0.4

Table S3 Statistical significance (p-value) between content of triacylglycerol and fatty acids composed triacylglycerol pool via gene combinations aimed at producing EPA (20:5$^{\Delta5,8,11,14,17}$) with and without co-expressed with *Pt*DGAT2b. Statistical significance was calculated in two-tailed Student’s t-test. Data regarding fatty acids composition of TAG for combination without *Pt*DGAT2b co-expressed presented in Klińska-Bąchor et al., 2024 (in review in Scientific Reports).

	*Ot*D6+PSE+*Tc*D5<br>vs<br>*Ot*D6+PSE+*Tc*D5+*Pt*DGAT2b	*Ot*D6+PSE+*Pt*D5<br>vs<br>*Ot*D6+PSE+*Pt*D5+*Pt*DGAT2b
TAG content	0.02	0.03
16:0	0.002	0.5
18:0	0.5	0.2
18:1$^{\Delta9}$	0.04	0.2
18:2$^{\Delta9,12}$	0.4	0.7
18:3$^{\Delta6,9,12}$	0.09	0.08
18:3$^{\Delta9,12,15}$	0.02	0.01
18:4$^{\Delta6,9,12,15}$	0.1	0.5
20:0	0.4	0.6
20:4$^{\Delta5,8,11,14}$	0.05	0.001
20:4$^{\Delta8,11,14,17}$	0.7	0.9
20:5$^{\Delta5,8,11,14,17}$	0.05	0.05

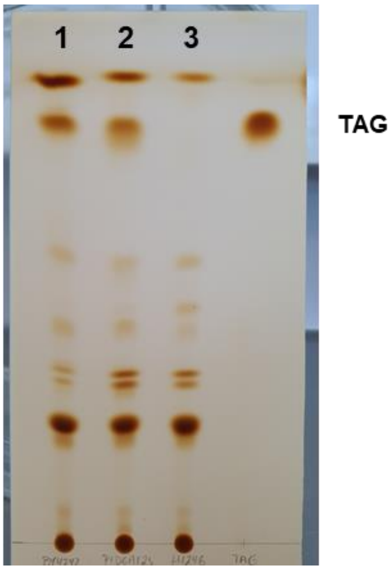

Figure S1 Complementation of triacylglycerol biosynthesis in *Saccharomyces cerevisiae* TAG-deficient mutant with the cloned *Pt*DGAT2b. The photograph presents a thin-layer chromatography (TLC) plate after separation of yeast extracts in hexane:diethyl ether:acetic acid (70:30:1) and stained in iodine vapors. From the left side: 1) BY4742 – wild yeast strain, positive control; 2) transformant of H1246 strain with *Pt*DGAT2b; 3) H1246 – mutant strain, unable to TAG production with incorporated pYES-DEST52.

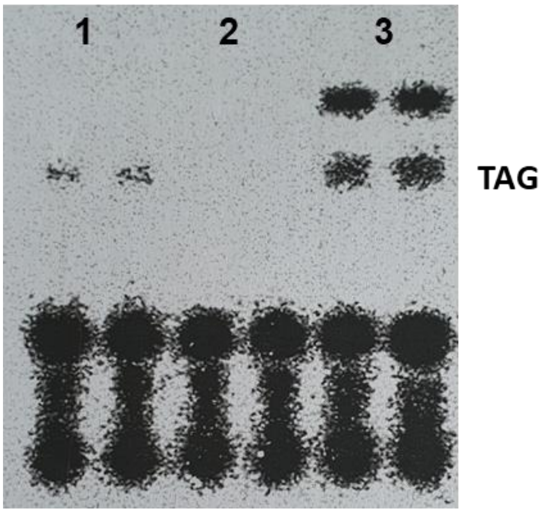

Figure S2 Triacylglycerol biosynthesis ability of *Pt*DGAT2b. The photograph presents a thin-layer chromatography (TLC) plate after separation of chloroform fraction of *in vitro* reaction mixture separated in hexane:diethyl ether:acetic acid (70:30:1) and visualized by autoimager. Activity determined in *in vitro* experiments with [^{14}C]-labeled acyl-CoA and unlabeled diacylglycerol for enzymes present in microsomal fraction derived from (from left side): 1) transformant of H1246 strain with *Pt*DGAT2b 2) H1246 – mutant strain, unable to TAG production, with introduced pYES-DEST52; 3) BY4742 – wild yeast strain, positive control.

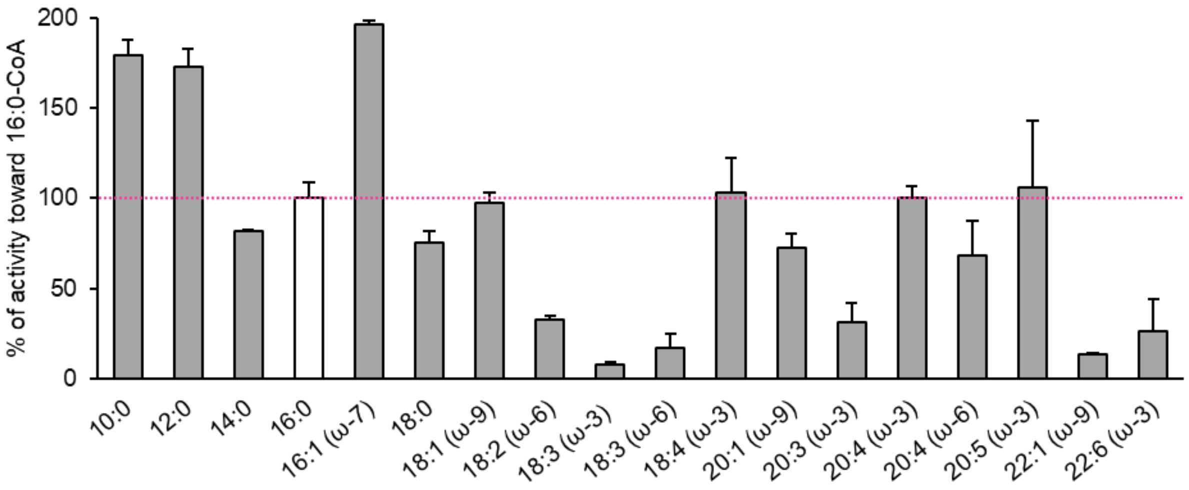

Figure S3 Preferences towards different acyl donors of *Pt*DGAT2b. As a reference activity, the activity detected toward 16:0-CoA was used. Error bars present standard deviations between independent biological replicates.
